# Group-based caregiver support interventions for children living with disabilities in low-and-middle-income countries: Narrative review and analysis of content, outcomes, and implementation factors

**DOI:** 10.7189/jogh.14.04055

**Published:** 2024-04-12

**Authors:** Christine He, Natalie Evans, Hamish Graham, Kate Milner

**Affiliations:** 1 Western Health, University of Melbourne, Victoria, Australia; 2 Melbourne Children’s Global Health, Murdoch Children’s Research Institute, University of Melbourne, Melbourne, Australia; 3 Melbourne Children’s Global Health, Murdoch Children’s Research Institute, University of Melbourne, Department of Pediatrics, Royal Children’s Hospital, Melbourne, Australia; 4 Neurodisability & Rehabilitation Research Group, Murdoch Children’s Research Institute, University of Melbourne, Department of Pediatrics & Department of Neurodevelopment & Disability, Royal Children’s Hospital, Melbourne, Australia

## Abstract

**Background:**

Caregivers of children with disabilities in low-and-middle-income countries (LMICs) face unique demands and challenges. Group education and support interventions can benefit parental and child well-being, however there is limited data from LMIC settings.

**Methods:**

We systematically searched databases (Medline, Embase, PsycINFO) for English language publications reporting on group-education and support-interventions for caregivers of children living with disability in LMICs, published between April 2012 and April 2022 using pre-established inclusion and exclusion criteria. Data from included studies was extracted and descriptively analysed in an online form, covering population, intervention, outcomes, and factors affecting implementation with key findings reported using narrative synthesis.

**Results:**

Of 1557 studies identified, 31 studies were included for full text review. The majority of these were qualitative (n = 15, 48%) and from middle-income countries (n = 26, 83.9%). A minority of studies were conducted in low-income-countries (n = 4, 12.9%). Recurring themes in caregiver support-intervention content include child behavioural management (n = 17, 54.8%) and child activities-of-daily-living (n = 16, 51.6%). Almost all interventions were facilitated by non-specialists (n = 23, 74.2%). Interventions mostly employed active learning strategies, including group discussion (n = 17, 54.8%) and hands on activities (n = 11, 35.5%). Outcomes across studies suggest interventions have the potential to improve many aspects of caregiver and child functioning. Major barriers to intervention implementation included lack of caregiver support from family members, time constraints on caregivers and poverty. The social support networks and education regarding childhood disability in a broader social context provided to caregivers during support group interventions reduce the social isolation and stigma experienced by caregivers of children living with disability in LMICs. Relevant appropriately targeted intervention content and supportive facilitators contributed to caregiver satisfaction toward support interventions. Caregiver support group interventions established in LMICs should prioritise sustainability through building strong partnerships with government and non-for-profit organisations.

**Conclusions:**

Caregiver support group interventions provide a promising avenue of improving caregiver and child outcomes for children living with disability in LMIC settings. More research is needed in this area.

Disability describes the complex interplay between health conditions, personal circumstances, and the environment, which results in impairment in bodily structure and function, limitation in activity or restriction in social participation [1]. Experience with disability is individualised, as disability encompasses a wide variety of conditions and a spectrum of condition severities [1]. This is especially true of disability in childhood which evolves and changes over time due to physical, psychological, and social changes over the life-course, with many impairments having substantial impacts on both the affected child and their family [2]. Children living with disability are more likely to have poorer health, education, and well-being compared to age-matched peers with normal development [2,3]. Caring for a child with disability can be associated with strain on family relationships, increased depression, anxiety, burnout, fatigue, social isolation, and prejudice [4–6].

Globally, an estimated 53 million children under the age of five years live with a disability, with 95% residing in low-and-middle-income-countries (LMICs) [2]. Previous studies have found a strong reciprocal association between poverty and disability, with poverty increasing the likelihood of disability and disability accentuating poverty [7]. Limited access to formal early childhood intervention services, education, health care, allied health, ancillary and social services in LMICs, also often leads to further disadvantage for families, with caregiving responsibilities typically falling upon caregivers without any professional guidance or support [8,9]. Given the inaccessibility of specialist disability services in many LMICs, there is a demand for research into alternative community-based approaches, which may be appropriate for providing increased support for children living with disability and their caregivers.

Support for caregivers of children with disability is vital in optimising well-being of children with disability and their families [3]. In high-income-countries (HICs) caregiver support and education interventions have demonstrated effectiveness in assisting parents of children with disability to cope positively in their daily caregiver responsibilities [10–12]. These interventions have led to improvements in adaptive parenting skills, parental well-being, and child behaviour [10–12]. Delivery of interventions in a group-based format may provide an additional benefit of providing social support to a vulnerable population, who often experience social isolation and discrimination from their communities [13,14]. Group-based interventions may also utilise fewer resources when upskilling and supporting caregivers [10]. Thus, it appears that caregiver support groups may be a promising avenue to support caregivers of children with disabilities in LMICs.

Despite established evidence on the effectiveness of group-based education and support for caregivers of children with disability in HICs, there is limited research on the implementation of caregiver support and education in LMIC settings. One previous review on support-groups in LMICs for intellectual-disability identified 13 studies dating back to 1989 [15], but no reviews have explored children with other neurodevelopmental disabilities (e.g. autism-spectrum-disorder (ASD), cerebral palsy) in LMIC settings. This review aimed to explore: a) content and composition of current group-based caregiver support and education interventions implemented in LMICs; b) impact of these interventions on caregiver and child outcomes; c) barriers and enablers to implementation of these interventions in LMICs.

## METHODS

### Search strategy

We searched online databases (Medline, PsycINFO and Embase) for articles published from April 2012 to April 2022, describing educational support-group interventions for caregivers of children living with disability. The search strategy was constructed based on previously published literature with a similar research focus [10,12] with search terms related to caregivers, children, disability, and group education (**Table 1**). A full search strategy is available in Table S1 in the **Online Supplementary Document**. The search was restricted to papers in English for feasibility and LMICs in keeping with our study question.

**Table 1:** Key search terms

Key Concept	Key words
Caregiver	Parent, carer, caregiver, mother, father, guardian
Child	Newborn, baby, neonate, infant, toddler, pre-schooler, kinder, boy, girl, child, childhood, paediatric, school-age, schoolchild, adolescent, youth, teen, teenage
Disability	Disability, impairment, deficit, disorder, neurodevelopmental disorder, brain injury, cerebral palsy, movement disorder, paralysis, congenital malformation, muscular dystrophy, spinal muscular atrophy, amyotrophic lateral sclerosis, spina bifida, myelomeningocele, spinal cord injury, intellectual disability, autism, fragile X, downs syndrome, chromosomal disorder, foetal, alcohol Spectrum Disorder, learning disability, Tourette, sensory disability, deaf, blind, visual impairment, hearing impairment
Group education and support	Psychoeducational support, support programme, group-based intervention, group therapy, psychosocial support, community-based rehabilitation, community-based inclusive development, self-help, community-health group, parent intervention, parenting programme, peer support

### Inclusion criteria

We included peer-reviewed studies reporting on any interventions for caregivers of children with disability in LMICs conducted in a group setting with an educational component. We defined educational component as any exercise aimed at providing instruction about disability, caretaking, or coping skills. Caregivers were defined as anyone with parenting or caring responsibilities for children up to the age of 18 living with disability. Studies could be condition-specific or non-condition specific and could be undertaken in any setting (e.g. community, health care service). We defined disability broadly, aiming to capture populations with neurodevelopmental, intellectual, and physical impairment, and including specific search terms (e.g. cerebral palsy, autism, Down Syndrome) for common impairments to maximise results. We used the World Bank classification for LMICs; including studies conducted in countries having a Gross National income (GNI)<13 205 US dollars per capita [16].

We excluded studies focusing on a single symptom or behavioural concern (e.g. sexual health of children with intellectual disability, feeding practices of children with cerebral palsy). We also, excluded interventions focused on chronic medical conditions (e.g. asthma, diabetes, cystic fibrosis, epilepsy) and behavioural or mental health disorders (e.g. attention deficit hyperactivity disorder, oppositional defiant disorder (ODD)).

### Data extraction and synthesis

We imported search results into an online review manager (Covidence, Melbourne, Australia). Two investigators (CH and NE) independently screened all titles and abstracts and then reviewed the full text of all potentially eligible articles for inclusion. Disagreement was resolved through discussion between CH and NE. A consensus was reached on all disagreements; so, an independent reviewer was not required.

CH completed the data extraction using a data extraction form on Covidence developed by the authors of this paper. The data extraction form included study objectives, intervention details including: group size, duration, location of support group, facilitator characteristics, parental outcomes, child outcomes. Qualitative data was analysed on a tabulated spreadsheet and main themes were identified. Quantitative data was analysed using R Studio. We selected support-intervention content and mode of delivery as the primary outcome due to its relevance to the study objective. Secondary outcomes of interest included primary effects of the support interventions on caregivers and their children, enablers and barriers to intervention implementation, facilitator characteristics and country of intervention implementation.

We categorised studies based on the primary population of focus and described the interventions narratively, with tabular summaries. We mapped all reported outcomes in tabular format for easy comparison, with narrative synthesis of findings. We synthesised reported barriers and enablers into themes, using tables and narrative description. We followed PRISMA reporting standards [17].

## RESULTS

**Figure 1:**
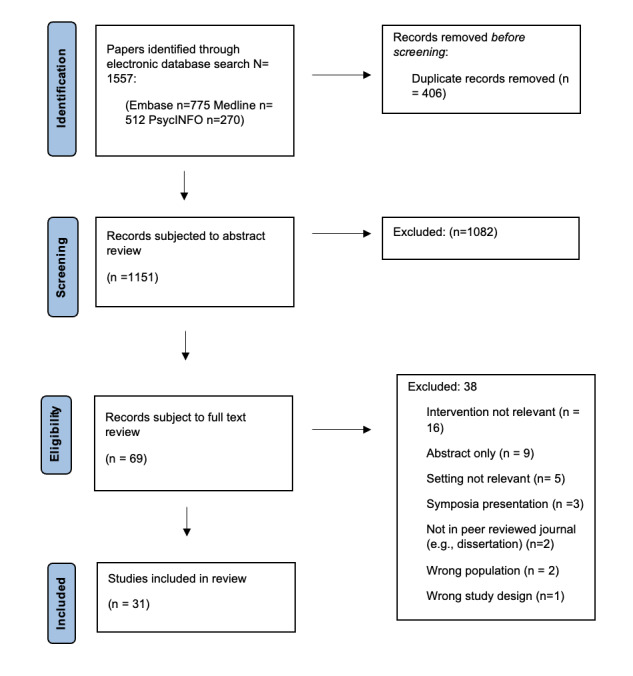
PRISMA flow diagram of study selection.

Our database search identified 1557 unique studies, 69 were subjected to full-text review and 31 met eligibility criteria and were included in qualitative synthesis (**Figure 1**).

### Study characteristics

**Figure 2:**
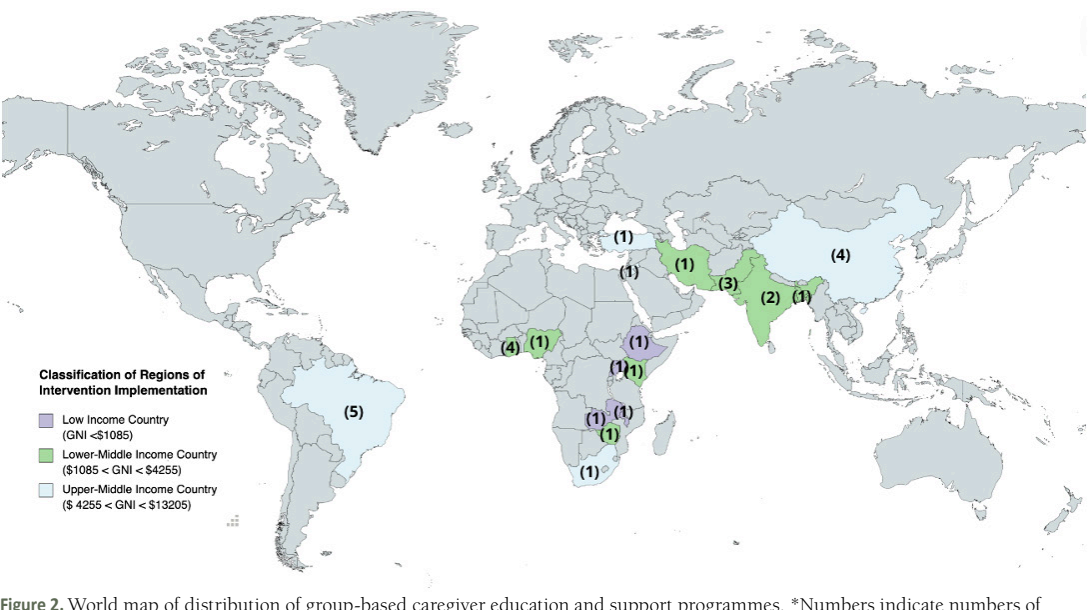
World map of distribution of group-based caregiver education and support programmes. *Numbers indicate numbers of included studies per country. One intervention was conducted across multiple unspecified regions and was not included in **Figure 2**.

Most of the included studies were qualitative (n = 13, 41.9%) or involved non-randomised quantitative comparisons (n = 12, 39%), with four included randomised controlled trials (12.9%) (**Table 2****,** Figure S1 in the **Online Supplementary Document**. Most included studies had a small sample size of less than 50 participants (n = 15, 48%). Nine studies (48%) had a moderate sample size between 50 and 100 participants, whilst four studies (13%) had a large sample size >200. A large proportion of identified studies were for caregivers of children with ASD (n = 8, 26%) or cerebral palsy (CP) (n = 7, 23%), or broadly defined neurodevelopmental disorders/delays (n = 7, 23%) (**Table 2****,** Figure S1 in the **Online Supplementary Document**). While age was not always specified (n = 8, 26%), five studies (16%) focussed exclusively on the early childhood period (aged under five years) while the remainder included older children and some included adolescents (**Table 2**, Figure S1 in the **Online Supplementary Document**). Most studies were conducted in UMICs (n = 12, 38.7%) [18–29] and LMICs (n = 14, 45.2%) [8,14,30–41]. Four studies (12.9%) [42–45] were conducted in LICs, and one study was conducted across multiple regions (**Figure 2**).

**Table 2:** Study characteristics

Author & country	Study design	Sample size	Setting	Population	Primary outcomes	Secondary outcomes	Key findings
Al-Khalaf et al., 2014, Jordan [20]	Qualitative research	10 mother-marital partner dyads	Lower-middle income country (LMIC)	Mothers of preschool age children with autism spectrum disorder (ASD) and the mothers’ marital partners	The provision of an education programme for mothers of preschool age children with ASD was associated with statistically significant improvement in coping skills and stress levels of mothers.	Mothers of preschool age children with ASD had higher stress levels and lower coping skills than their partners.	Education programmes for Jordanian mothers of children with ASD can help promote optimal development in young children with a disability, which may facilitate greater social and occupational participation amongst children with disability when they reach adulthood.
Bello-Mojeed et al., 2016, Nigeria [31]	Feasibility study	20 children and their respective mothers	Lower-middle income country (LMIC)	Children <19 y old with ASD and a history of aggressive and self-injurious behaviour attending the Neurodevelopmental Clinic at Child and Adolescent Mental Health Service Unit of Federal Neuro-Psychiatric Hospital and their respective mothers.	Parent delivered behaviour interventions are feasible to implement in resource-poor settings like Nigeria if the programme is tailored to the education level and health literacy of parents	Nil reported secondary outcomes	Parent delivered behaviour intervention was associated with significant reduction in the challenging behaviour of children with ASD
Bordini et al., 2020, Brazil [21]	Randomised controlled trial	66 families	Upper-middle-income country (UMIC)	Children with ASD aged between 3 and 6 y and 11 mo with an IQ score between 50 and 70 and their caregivers.	Parent training using video modelling led to statistically significant improvements in the IQ of their children with ASD (I Q Cohen’s d = 0.433, *P* < 0.001).	Parent training using video modelling did not have statistically significant effects on communication, social functioning in children with ASD.	Caregivers’ mental health may have a direct effect on adherence and effectiveness of parent led interventions
Brezis et al., 2015, India [32]	Qualitative research	39 children and their respective primary caregiver (i.e. mother or father)	Lower-middle income country (LMIC)	Families with children with ASD between the ages of 2.3 and 10.6	Following parental training, parents had a more positive optimistic perception of their children with ASD and were less likely to compare their children with ASD to neurotypical peers or siblings.	Parents who came from relatively lower income families were more preoccupied by their child’s ability to live independently and had greater concerns for what would happen to their child following their death.	Following parental training there was greater parental acceptance of their child’s diagnosis and reduced prioritisation of societal approval
Bunning et al., 2020, Kenya [14]	Qualitative research	81 caregivers	Lower-middle income country (LMIC)	Caregivers of children aged 0–15 y of age with general disability in Kilifi, Kenya	Empowering self-help groups for caregivers of children with disabilities are associated with significant growth in caregiver report of their social support networks, reduced perception of the severity of their child’s condition.	Self-help groups led to improvements in the well-being of caregivers by facilitating social ties and support, leading to a greater sense of agency and capacity for caregivers.	Self-help groups for caregivers of children with disabilities in lower-resourced settings enable caregivers to take control of their own lives and the lives of their children.
Cenk et al., 2016, Turkey [29]	Quasi-experimental	104 caregivers	Upper-middle-income country (UMIC)	Caregivers of children with intellectual and physical disability attending specialised education and rehabilitation institutions in the Kötekli region	Education programmes for families with intellectually disabled children were effective in improving the knowledge caregivers had about their child’s disability	Education programmes for families with children with intellectual disability led to significant reduction in hopelessness	Parental education contributes to long term improvements in intrafamilial communication, coping with stress and social functioning for families of children living with intellectual disability. Future family-centred education programmes which provide an opportunity for families to share their emotions, experiences and problems may be beneficial.
Dambi et al., 2016, Zimbabwe [37]	Cross-sectional study	49 caregivers	Lower-middle income country (LMIC)	Primary and informal caregivers of children (ages between 3 mo and 12 y) with cerebral palsy who had either attended CP workshops or had not attended such workshops.	Caregivers who attended an educational workshop had a greater knowledge about the definition of CP, however findings were not corrected for confounders	Nil secondary outcomes	It is recommended for health care professional to make deliberate efforts using structured interventions (formal workshop curricula) to educate caregivers about CP.
Donkor et al., 2019, Ghana [35]	Qualitative research	16 caregivers	Lower-middle income country (LMIC)	16 caregivers of children (ages 1–16 y) with CP registered in a community-based rehabilitation programme.	Caregiver interventions can alleviate some burdens faced by caregivers in relation to feeding, however interventions solely involving caregiver training were unable to lead to significant improvements in children’s anthropometric nutritional status.	Nil secondary outcomes	Improved utilisation and effectiveness of mainstream therapeutic feeding programmes may be required alongside caregiver training to improve the anthropometric nutrition status of children with CP
Duttine et al., 2019, Brazil [25]	Pilot study	Not applicable	Upper-middle-income country (UMIC)	Caregivers of children with Congenital Zika Syndrome in Brazil	The Juntos programme (an adaptation from the GTKCP programme) to support caregivers of children with congenital zika syndrome was found to be a feasible and acceptable intervention.	Nil secondary outcomes	There are gaps in service provision for caregivers of children with CZS living in Brazil
Duttine et al., 2020, Brazil [26]	Pilot study	48 families	Upper-middle-income country (UMIC)	Caregivers of children (<18 y) who have confirmed or suspected CZS but no other types of neurodevelopmental disabilities.	Caregivers who participated in the Juntos community support group intervention expressed comfort in seeking solidarity with co-participants who had undergone similar experiences	Nil secondary outcomes	Juntos has the potential to be an important resource for community practice and there is a scope to expand across Brazil and in other South American countries and to children with other developmental disabilities.
Fang et al., 2022, China [18]	Qualitative research	14 caregivers	Upper-middle-income country (UMIC)	Mothers and grandmothers of children (aged 3–6) with ASD	Caregivers found the Stars and Rain Education Institute for Autism (SEIRA) parent training programme to be helpful and informative.	Following SEIRA programme participation, caregivers report improved subjective assessments of mental well-being, emotional relationships with their child and partner.	In terms of programme design, adequate opportunities for caregivers to practice and receive feedback, as well as a mixture of group and individualised support, are central to creating an overall positive and empowering experience.
Habib-Hasan et al., 2020, Pakistan [41]	Feasibility study	48 families	Lower-middle income country (LMIC)	Parents of children (aged 0–5 y) with Down Syndrome	Parent Empowerment Programs for children under 5 y of age with Down Syndrome empowered parents to provide developmental and motor skill building activities and was perceived to be worthwhile by families.	Parent Empowerment Program for caregivers of children living with Down Syndrome was effective in improving gross motor function in children with downs syndrome in a resource poor setting.	Expanding the Parent Empowerment Program for caregivers of children living with Down Syndrome through ‘train the trainer’ models can be an economically viable training system, which would engage the parents of children with DS, promote community participation and improve the functional outcomes for children with Down Syndrome.
Hamdani et al., 2015, Pakistan [39]	Pilot study	68 caregivers	Lower-middle income country (LMIC)	Families of children with developmental disorders	Parental interventions led to significant improvement in child physical and social functioning, reduced stigmatisation, and greater family empowerment to seek community services for the child.	Caregivers did not experience increased burden following intervention participation	Cascade models of intervention delivery has the potential to be self-sustaining, offering an innovative solution for supporting families with children with developmental disabilities in lower resourced settings.
Hamdani et al., 2021, Pakistan, [40]	Randomised controlled trial	540 parent-child dyads	Lower-middle income country (LMIC)	Parent-child dyads where the child had a developmental disability (e.g. intellectual disability, motor difficulties, speech and communication difficulties or Down syndrome)	Technology- assisted, family-volunteer delivered, brief, multicomponent, parents’ skills training did not improve child functioning beyond those in the enhanced treatment as usual arm (DD-CDAS change −2.63 *P* = 0.1820).	Technology-assisted, family-volunteer delivered, brief multicomponent parents’ skills training led to significant improvements in caregivers’ quality of life (PedsQL total score difference 5.35, *P* = 0.0268)	Digital technology has a potential to deliver parent-mediated interventions, especially to scale up interventions in low resource settings.
Hearst et al., 2020, Zambia [45]	Pilot study	129 caregiver-child dyads	Low Income Country (LIC)	Parents/ guardians and a child with disability living in two low-income, high-density compounds in Zambia	Kusamala + community-based intervention did not lead to significant reductions in parent and community stigma towards children with disabilities.	Caregivers and members of the community perceived children with disability to experience less rejection from their peers and families following the Kusamala + community-based intervention, however there was an increased perception that families should feel ashamed and reduced perception that children with disability should be treated the same as age-matched neurotypical peers.	Community-based interventions are acceptable and feasible for families with children with disabilities, however government support and prioritisation of children with disabilities is essential in ensuring the success of these initiatives.
Karim et al., 2021, Bangladesh [36]	Quasi-experimental	134 caregiver-child dyads	Lower-middle income country (LMIC)	Primary caregivers and their child with cerebral palsy attending CSF ‘Shishu Shorgo’ Rehabilitation and Early Intervention Centre	Community based intervention led to significant improvements in motor function, communication, and speech in children with cerebral palsy.	Community based interventions did not lead to significant improvements in caregiver depression or anxiety	Community based interventions provides sustainable support for children with cerebral palsy and their caregivers in low-resourced settings where trained health workers may be scarce.
Leung et al., 2013, China [24]	Randomised controlled trial	81 parents	Upper-middle-income country (UMIC)	Parents of preschool aged children with confirmed diagnoses of neurodevelopmental disorder who were attending professional rehabilitation services.	The Triple P programme was efficacious and acceptable to Chinese parents with preschool aged children with neurodevelopmental disability	The Triple P programme led to reductions in child problem behaviour (ECBI-Problem, t(79) = 3.21, *P* = 0.002), parental stress (PSS, F(1,78) = 15.60 − 25.86, *P* < 0.001), parental conflict (PPC-Intensity, F(1, 78) = 19.99–22.31, *P* < 0.001, PPC-Concern, F(1, 78) = 22.47–23.94, *P* < 0.001), and parental dysfunctional discipline styles (PS-Laxness, F(1, 78) = 4.46–7.13, *P* = 0.038–0.009, PS-Over-reactivity, F(1, 78) = 5.85–8.81, *P* = 0.018–0.006)	The Triple P programme is effective in supporting the needs of Chinese families with children with developmental disabilities, in a social context where there may be stigma and shame associated with disability.
Masulani-Mwale et al., Malawi [44]	Feasibility study	Not specified	Low-income country (LIC)	Parents of children with intellectual disabilities in Malawi	The Titikulane psychological support intervention for parents of children living with intellectual disability was perceived to be feasible and useful for parents of children with an intellectual disability	Parents of children with intellectual disability identified child challenging behaviours, community perception and stigma, marital issues, stress management, respite services and coping strategies as important topics to address in a targeted parental support intervention.	Community based group intervention is efficacious in supporting parents of children with intellectual disability in low resourced regions and can reduce poverty for these populations
McDevitt, 2021, China [19]	Qualitative research	294 parents	Upper-middle-income country (UMIC)	Parents of children (aged 2-7 y old) diagnosed with ASD.	Despite initial difficulties for parents to engage with online group parent education programmes, they greatly benefited from the cultivated support networks, which parents perceived to be essential in helping them overcome parenting challenges during the COVID19 pandemic.	Nil secondary outcomes	Training of more teachers to engage with children with ASD and their parents and the provision of culturally relevant instructional materials will contribute to the successful implementation of parental support initiatives for parents of children with ASD in China
Muthukaruppan et al., 2020, India [38]	Cohort study	308 caregivers	Lower-middle income country (LMIC)	Primary caregivers of children with developmental delays (aged 0 − 7 y old) enrolled in ASSA’s Village-based Early Intervention Program	Community-based early intervention programme for caregivers of children with developmental delays was associated with significant improvement in caregiver strain and family empowerment.	Nil secondary outcomes	Education, support, and training for caregivers of children with developmental delays using a family centred approach may be an important component of early intervention therapy for these families
Pashazadeh Azari et al., 2019, Iran [30]	Randomised controlled trial	31 parents	Lower-middle income country (LMIC)	Parents of children (aged 3 − 10 y old) with ASD	CI-ASD (Contextual Interventions for ASD) parental group training led to improvements in children’s social participation in family activities (COPM performance effect size = 0.397, *P* < 0.001) and parental efficacy (PSEM effect size = 0.144, *P* = 0.013) in caring for a child with ASD	Parents perceived CI-ASD to be an acceptable intervention	The CI-ASD programme is efficacious in eliminating children’s sensory issues, promoting increased social participation of children with ASD and improving parental outcomes.
Sadoo et al., 2022, Uganda [43]	Quasi-experimental	48 families, 93 community health care workers	Low Income Country (LIC)	Families with children (aged 0 − 3 y old) with early developmental disabilities in Uganda, Nursing/midwifery staff working in Ugandan community health care centres	A family centred programme aimed towards early detection and intervention for children with developmental disability was feasible and acceptable in a rural sub-Saharan African setting.	Nursing/midwifery staff involved in community based early intervention training perceived training to be feasible and helpful in improving knowledge and increasing confidence in supporting children with early developmental disabilities and their caregivers.	Community based group early intervention programmes have the potential to improve familial quality of life, however barriers to implementation including programme accessibility, poverty, gender inequality and stigma should be addressed in lower-resourced settings to facilitate greater programme outreach and engagement.
Salomone et al., 2019 Multiple LMICs in Africa, Americas, Eastern Mediterranean, Europe, Southeast Asia, Western Pacific (specific countries not specified) [68]	Qualitative research	Not applicable	Range of regions	Not applicable	Identified themes important to cover in a caregiver mediated intervention for neurodevelopmental disorders and delays include behavioural management, management of medical complications, activities of daily living, communication, play and leisure, parent emotional regulation/mindfulness/stress reduction.	Adapting WHO-CST to different cultural contexts requires explanation of technical terms at a literacy level appropriate for intended participants, incorporating local stories and examples, as well as providing culturally appropriate additional activities	Additional studies evaluating cost-effectiveness, component analysis, alternative delivery methods and optimal programme duration and frequency would be beneficial in guiding future programme implementation
Smythe et al., 2019, Brazil, [27]	Qualitative research	49 families	Upper-middle-income country (UMIC)	Fathers of children with neurologist confirmed Congenital Zika Syndrome (CZS)	Fathers of children with CZS perceived the group format of Juntos to be acceptable and offered them the opportunity to share experiences of caring for their child.	Clear goals of parental involvement in group interventions and kindness of facilitators were associated with increased engagement of fathers with the Juntos programme, whilst work commitments and cultural norms were identified barriers of paternal engagement	Identifying strategies to involve fathers during intervention development stages, clearly communicating goals/expectations of the programme and providing more tailored materials for fathers can help improve paternal engagement with parental support interventions.
							
Smythe et al., 2020, Brazil [28]	Qualitative research	30 caregivers	Upper-middle-income country (UMIC)	Caregivers of children with neurologist confirmed Congenital Zika Syndrome (CZS)	Expert mothers as facilitators for a community-based group intervention for children with CZS and their caregivers was perceived to be acceptable by caregivers	Budling a social support network for caregivers of children with neurodevelopmental disability through group community-based interventions can improve the knowledge and skills of caregivers and provide support through the creation of a sense of belonging	Caregivers with similar life experiences may provide innovative community support to families of children with CZS in resource limited contexts
							
Tekola et al., 2020, Ethiopia, [69]	Qualitative research	2 Ethiopian Master trainers, 9 caregivers, 5 programme facilitators/observers	Low-income country (LIC)	CST programme master trainers, facilitators, observers, and participants	Caregivers who participated in the WHO CST intervention perceived the programme to be useful, leading to improved psychological well-being and knowledge/skills regarding caring for their child	CST programme facilitators and observers perceived caregivers to have a more optimistic perspective of their child’s development following WHO CST participation	Inclusion of topics on toilet training and protecting children from abuse and providing more support to caregivers with lower health literacy may improve the WHO-CST intervention.
van Aswegen et al., 2019, South Africa, [23]	Quasi-experimental	18 caregivers	Upper-middle-income country (UMIC)	Caregivers of children (aged 0-8 y old) with cerebral palsy	Participation in Hambisela (a group-based education intervention for caregivers of children with CP) was associated with no significant differences in caregiver stress levels or total quality of life scores.	Higher levels of education amongst caregivers were associated with lower stress levels. There was no correlation between the child’s age, level of impairment, caregiver’s age or employment status and level of caregiver stress.	Identifying specific factors contributing to caregivers’ stress levels and poor QOL in a South African context can assist in the development of an effective support programme for caregivers of children with CP.
Zu et al., 2019, China [22]	Quasi-experimental	16 mothers	Upper-middle-income country (UMIC)	Mothers with one child (aged 4-20 y old) diagnosed with ASD in Shanghai and Taichung	Following participation in a psychoeducational group intervention mother of children with ASD in both Shanghai and Taichung utilised more positive language when referring to their child	Participation in a psychoeducational group intervention was associated with reduced depression in mothers of children with ASD and reduced ASD symptoms in children	An effective psychoeducational support intervention for mothers of children with ASD should include professional support, opportunities for mothers to vent their emotions and adequate support in navigating family relationships.
Zuurmond et al., 2018, Ghana [8]	Qualitative research	64 caregiver child dyads	Lower-middle income country (LMIC)	Caregivers of children (aged 18 mo to 12 y) with cerebral palsy	Traditional beliefs of disability being associated with the supernatural in low resourced settings, stigma/discrimination, lack of support for caregivers and the physical burden of caring for a child with disability influence the outcomes of caregivers of children with cerebral palsy in a Ghana context.	Participation in the GTKCP parental support group intervention is associated with improved overall caregiver quality of life through improved knowledge, reduced stigma, and increased optimism towards their child’s outcomes.	A community-based support intervention which adequately engages the caregiving dyad, broader extended family and community will be effective in reducing the effect of childhood disability on household poverty and ameliorating stigma/discrimination.
Zuurmond et al., 2019, Ghana [33]	Qualitative research	64 caregiver-child dyads	Lower-middle income country (LMIC)	Caregivers of children (aged 18 mo to 12 y) with cerebral palsy	Participation in GTKCP parent training programme was associated with significant improvements in caregiver, QOL, knowledge and attitudes towards caring for the child.	Participation in GRKCP parent training programme was associated with significant improvements in caregiver’s perception of their child’s physical and emotional health, however, was not associated with any objective improvements in child nutritional status.	Caregiver empowerment programmes can lead to sustainable change for parents of children with cerebral palsy however nutrition/feeding of children should be more greatly prioritised in such interventions
Zuurmond et al., 2020, Ghana, LMIC [34]	Qualitative research	18 caregivers	Lower-middle income country (LMIC)	Caregivers of children (aged 18 mo to 12 y) with cerebral palsy	The process of caregiver empowerment following participation in GTKCP parental training is multifaceted and involves the individual, family structure and socioeconomic determinants of health	GTKCP participation encouraged greater caregiver engagement with advocacy for children with disability in the local community.	Interventions which place too much emphasis on individual agency and improved self-efficacy are unrealistic in low-resourced settings. A more efficacious intervention needs to address familial and socio-political issues, which act as barriers to caregiver empowerment.

### Content and facilitators of group-based support interventions

#### Intervention content

Detailed descriptions of intervention content and materials were included in 20 studies (64.5%) [8,18–23,25–29,31,33,38,40,41,44,46,47]. Detailed intervention descriptions were linked in the supplemental material of five studies (16.1%) [30,32,35,36,43] and six studies (19.4%) [24,34,37,39,42,45] did not include a detailed intervention description. 22 studies (71.0%) [8,18,20–23,29,31–34,36–41,43–47] described completely new interventions and nine studies (29.0%) [19,24–28,30,35,42] described interventions adapted from previously established interventions.

The different interventions identified by our review varied greatly in terms of the content they included, who facilitated the groups, how frequently and for how long they met, and what activities they did together (**Table 3**). The intervention content and design of identified studies were generally tailored to the disability type (e.g. Cerebral Palsy, Congenital Zika syndrome) and influenced by the sociocultural context (e.g. political, and cultural beliefs), with negligible difference attributable to broad country wealth classification or geographical location. Despite differences between caregiver support-groups, there were some recurring themes in the content of the support group interventions identified in our review, with more than half of identified interventions targeting child behavioural management (n = 17, 54.8%) [8–20,22,24–31,38,40,42,44,47] and child activities of daily living (ADLs) (n = 16, 51.6%) [8,19,23,25–29,33,34,36,40,41,43,45,47] (**Table 3**). Other recurring topics covered include information about child’s disability (n = 14, 45.2%) [20,22–29,31,37,38,40,43], advocacy for children with disability (n = 13, 41.9%) [8,14,22,25–29,33,34,38,39,44], child play and leisure (n = 13, 41.9%) [8,18,23,25–28,33,34,40,41,43,47], child development (n = 11, 35.5%), feeding practices (n = 11, 35.5%) [8,23,25–28,33,34,39,43,45], parenting skills (n = 9, 29.0%) [8,19,21,24,33,34,39,42,43], and parental stress reduction strategies (n = 9, 29.0%) [18,19,22,29,38,40,42,44,47] (**Table 3**).

**Table 3:** Summary of composition and implementation of caregiver support group interventions organised by primary disability of child

Author & Country	Programme Name	Duration	Location	Facilitator	Group size	Mode of delivery	Topics covered	Teaching methodology
**Autism**								
Fang et al., 2022, China [18]	SREIA (Stars and Rain Education Institute for Autism)	2 h, daily, 5 d a week for 11 consecutive weeks	Routine services for families of autistic children	Social workers, early education workers	10 caregivers	In-person group, online social media group, individual counselling, printed materials/manual	Behavioural management, child development, communication, play and leisure, parent emotional regulation/ mindfulness/ stress reduction, future planning	Group discussion, didactic lecture, group problem solving activities, storytelling, demonstration, homework and feedback, practical activities
McDevitt, 2021, China [19]	PET (Parent education and training programme)	12 weeks duration, length of psychoeducational components not specified	We-chat/ ding-talk smart phone applications	Cascade training model (head coach trained six supervisors who trained eight non-specialist volunteers, qualifications of head coach and supervisors not specified)	6 parents	Online social media group	Behavioural management, parenting skills, management of medical complications, activities of daily living, parent emotional regulation/ mindfulness/ stress reduction	Group discussion, demonstration, homework and feedback, practical activities, video
Pashazadeh Azari et al., 2019, Iran [30]	CI-ASD (Contextual intervention adapted for autism spectrum disorders)	2 group sessions of unspecified length, 10 weekly 45 min individual coaching sessions	Group intervention location not specified. Individual counselling occurred in participants’ home	Occupational therapist	Not specified	In-person group, individual counselling, printed materials/manual	Behavioural management, peer support, child emotional regulation, child health/functioning	Didactic lecture, homework and feedback, training/coaching
Al-Khalaf et al., 2014, Jordan [20]	Unnamed programme	4 h, weekly, 5 weeks duration	Private clinic	Psychologist	10 parents	In-person group	Behavioural management, information about child’s condition, communication, personal experiences	Group discussion, didactic lecture
Bello-Mojeed et al., 2016, Nigeria [31]	Unnamed programme	Unspecified length, weekly, 5 weeks duration	Hospital outpatient clinic	Consultant psychiatrist specialising in Child and Adolescent Mental Health	10 mothers	In-person group	Behavioural management, information about child’s condition	Group discussion, group problem solving activities
Bordini et al., 2020), Brazil [21]	Unnamed programme	1.5 h, weekly, 22 weeks duration	Hospital	Cofacilitated by two psychologists and one certified Applied Behavioural Analysis therapist	10–12 caregivers	In-person group, printed materials/ manual, audio/visual aids	Parenting skills	Practical activities, video viewing
Brezis et al., 2015, India [32]	PCTP (Parent-Child Training Program)	Unspecified length, unspecified frequency, 3 mo duration	Not specified	Expert mother	Not specified	In-person group	Not specified	Group discussion, practical activities
Zu et al., 2019 China [22]	Unnamed programme	1.5 h, weekly, 12 weeks duration	Mental health centre, university	Not specified	Not specified	In-person group	Behavioural management, information about child’s condition, management of medical complications, navigating community resources (education/disability rights/ health care), advocacy, communication, parent emotional regulation/ mindfulness/ stress reduction, peer support, child health/functioning, parental expectations, child emotional regulation	Not specified
**Cerebral palsy**								
Zuurmond et al., 2018, Ghana [8]	GTKCP (Getting to Know Cerebral Palsy)	3 h, monthly, 11 mo duration	In community	Cofacilitated by one physiotherapist or physiotherapy assistant and one primary health care worker (i.e. special needs teacher, nutritionist, community-based rehabilitation worker)	8–10 caregivers	In-person group; home visit	Parenting skills, navigating community resources (education/disability rights/ health care), activities of daily living, advocacy, communication, play and leisure, feeding, community inclusion, caring for child	Not specified
Zuurmond et al., 2019, Ghana [33]	GTKCP (Getting to Know Cerebral Palsy)	2 h, monthly, unspecified duration of programme	Not specified	Cofacilitated by one physiotherapist or physiotherapy assistant and one primary health care worker	8–10 caregivers	In-person group	See above	Not specified
Zuurmond et al., 2020, Ghana [34]	GTKCP (Getting to Know Cerebral Palsy)	3–4 h, monthly, 12-mo duration	Presbyterian Church of Ghana and other community sites	Cofacilitated by one physiotherapist and one primary health care worker (i.e. special needs teacher, nutritionist, community-based rehabilitation worker)	8–10 caregivers	In-person group	See above	Not specified
Donkor et al., 2019, Ghana [35]	GTKCP (Getting to Know Cerebral Palsy)	Not specified	Community site	Not specified	Not specified	In-person group	Not specified	Not specified
van Aswegen et al., 2019 South Africa [23]	Hambisela	3 h, weekly, over period of 8 weeks	Not specified	Not specified	8–10 caregivers	In-person group	Information about child’s condition, child development, activities of daily living, communication, play and leisure, feeding, caring for child	Group discussion, role play, demonstration, practical activities, viewing of video
Karim et al., 2021 Bangladesh [36]	Unnamed programme	3 h, daily, 5 d a week for 6 mo	Rehabilitation centre	Two community volunteers who have been trained by qualified physiotherapist, supervised by physiotherapist.	10 caregiver child units	In-person group and home visit	Chid development, activities of daily living, communication, peer support, social skills, movement, cognition	Group discussion
Dambi et al., 2016, Zimbabwe [37]	Unnamed programme	3-d intervention, duration of intervention unspecified	Hospital	Physiotherapists, occupational therapists, counsellors, rehabilitation technician, nurses, doctors	Not specified	In-person group and individual counselling	Information about child’s condition, personal experiences	Group discussion, didactic lecture, practical activities
**Neurodevelopmental disorders/delay**								
Salomone et al., 2019 Multiple LMICs in Africa, Americas, Eastern Mediterranean, Europe, Southeast Asia, Western Pacific (specific countries not specified) [68]	WHO CST (Caregiver Skills training)	2–2.5 h, unspecified frequency, 9 total core sessions and 7 optional sessions	Community centres, health centres and schools. Home visits in participants’ home	Non specialist volunteers	Not specified	In-person group, home visit, printed materials/manual	Behavioural management, management of medical complications, activities of daily living, communication, play and leisure, parent emotional regulation/mindfulness/stress reduction	Group discussion, role-play, case-based discussion, demonstration, didactic lecture, training/coaching
Tekola et al., 2020 Ethiopia [69]	WHO CST (Caregiver Skills training)	2–2.5 h, unspecified frequency, 9 total core sessions and 7 optional sessions	Child mental health clinic in hospital, community centres, health centres and schools. Home visits in participants’ home	One specialist (career not specified) and two non-specialist volunteers	Not Specified	In-person group, home visit, printed materials/manual	Behavioural management, parenting skills, communication, parent emotional regulation/mindfulness/stress reduction, child health, child development, child intelligence	Group discussion, role-play, case-based discussion, demonstration, didactic lecture, training/coaching
Sadoo et al., 2022 Uganda [43]	Baby Ubuntu early intervention programme	2–3 h, weekly/fortnightly, 6 mo	Child development centres. Home visits in participants’ home	Expert parent (parent of child with disability)	6–10 caregivers	In-person group, home visits	Parenting skills, information about child’s condition, activities of daily living communication, play and leisure, feeding, community inclusion, child mobility, caring for child	Not specified
Muthukaruppan et al., 2020, India [38]	mVBR-EI (Mobile Village Based Rehabilitation-Early Intervention)	2-d psychoeducation component, duration of other intervention components not specified	In villages	Community rehabilitation workers, consultant psychologist, rehabilitation specialist, parental peers	Not specified	In-person group, home-visit, telephone follow-up, online social media group, individual counselling, printed materials/manual, audio/visual aids	Behavioural management, information about child’s condition, advocacy, parent emotional regulation/mindfulness/stress reduction, community inclusion, caring for child	Not specified
Leung et al., 2013, China [24]	Triple P	2 h, 6 sessions over unspecified period +2 telephone follow up	Not specified	Social workers	Not specified	In-person group, telephone follow-up	Behavioural management, parenting skills, information about child’s condition, parental expectations	Group discussion, didactic lecture, role play, homework and feedback, practical activities
Hamdani et al., 2015 Pakistan [39]	Unnamed programme	6-mo duration, length and frequency of sessions not specified	Not specified	Cascade network facilitation system (Trainer with master’s degree in psychology trained community volunteers who facilitated programme)	5–7 families	In-person group	Parenting skills, child development, advocacy, feeding, community inclusion, child physical health	Group discussion, role play, practical activities, video viewing
Hamdani et al., 2021, Pakistan [40]	Unnamed programme	1.5 h, weekly, 9 weeks duration	Not specified	Cascade network facilitation system (one master trainer with 16 y education and one year experience working with children with developmental disorders trained 10 trainers who had at least a master’s degree in clinical psychology trained community volunteers who facilitated programme)	4–5 families	In-person group	Behavioural management, information about child’s condition, management of medical complications, activities of daily living, play and leisure, parent emotional regulation/mindfulness/ stress reduction, peer support	Not specified
**Congenital zika syndrome**								
Duttine et al., 2019, Brazil [25]	Juntos	4 h, weekly, 10 weeks duration	Local community facilities including local health centres, offices of local organisations and schools	Cofacilitated by one expert mother and one allied health professional (physiotherapist)	Not specified	In-person group	Behavioural management, information about child’s condition, navigating community resources (education/disability rights/health care), child development, activities of daily living, advocacy, communication, play and leisure, feeding, community inclusion, child mobility, caring for child	Group discussion, demonstration, self-reflection
Duttine et al., 2020, Brazil [26]	Juntos	4 h, weekly, 10 weeks duration	Local community facilities including local health centres, offices of local organisations and schools	Cofacilitated by one expert mother and one allied health professional (physiotherapist)	Variable	In-person group	See above	See above
Smythe et al., 2019, Brazil, [27]	Juntos	4 h, weekly, 10 weeks duration	Not specified	Cofacilitated by one expert mother and one allied health professional (physiotherapist, occupational therapist or speech therapist)	Not specified	In-person group	See above	See above
Smythe et al., 2020, Brazil, [28]	Juntos	4 h, weekly, 10 weeks duration	In community	Cofacilitated by one expert mother and one allied health professional (physiotherapist, occupational therapist, or speech therapist)	8–10 caregivers	In-person group	See above	See above
**Intellectual disability**								
Masulani-Mwale et al., 2019, Malawi [44]	Titukulane	Not specified	Not specified	Not specified	14 parents	In-person group, individual counselling	Behavioural management, navigating community resources (education/disability rights, health care), child development, advocacy, parent emotional regulation/ mindfulness/ stress reduction, peer support, community inclusion, parental expectations	Not specified
Cenk et al., 2016 Turkey [29]	Unnamed programme	1.25–1.5 h, weekly, 3 weeks	Private rehabilitation clinic	Not specified	15–20 parents	In-person group	Behavioural management, information about child’s condition, management of medical complications, activities of daily living, advocacy, communication, parent emotional regulation/ mindfulness/ stress reduction, child safety	Didactic lecture, role play, storytelling, question/answer, practical activities
**Non-specific disability**								
Hearst et al., 2020, Zambia [45]	Kusamala +	Length unspecified, weekly, unspecified duration	Maternal child health clinics, local churches, home visits to participants’ home	Facilitators are community volunteers, supervised by health professionals including nurses, physiotherapists, dieticians, environmental health workers, members, members of the Zambian Association of Persons with Disabilities (ZAPD), administrators in the Ministry of Education and Social Welfare	20–25 child parent units	In-person group, home-visit, rehabilitative therapy	Child development, activities of daily living, feeding	Play therapy
Bunning et al., 2020, Kenya [14]	Unnamed programme	1 h, monthly, 6 mo, weekly peer support group meetings without facilitator	Community based	Community health workers	11 caregivers	In-person group	Navigating community resources (education/ disability rights/ health care), advocacy, personal experiences, economic empowerment, peer support, community inclusion	Group discussion
**Down syndrome**								
Habib-Hasan et al., 2020, Pakistan [41]	PEP (Parent Empowerment Program)	Length unspecified, 1–4 X monthly, 14 mo	Non-government organisation	Paediatric therapist	3–4 caregivers	In-person group, online social media group	Activities of daily living, play and leisure	Didactic lecture, demonstration, training/coaching, video viewing

#### Intervention duration

The frequency of support group convenance and duration of support group interventions varied greatly across studies. The duration of support group sessions ranged from one to four hours. The mean duration of included support group interventions was 2.82 hours (SD = 1.15 hours). Eleven studies did not include information on support group duration and were not included in calculation of mean intervention duration [19,30–32,35,37–39,41,44,45]. The shortest-term intervention was two days [38] and the longest-term intervention spanned 14 months [41]. The median length of support group interventions was 10.5 weeks. Two support groups convened five days a week [18,36], 16 support groups convened weekly [14,19–23,25–31,40,43,45] and four support groups convened monthly [8,33,34,41]. The frequency of support groups was unspecified or not applicable (i.e. short-term intervention) in nine studies [24,32,35,37–39,42,44,47].

#### Group facilitators

The ‘Baby Ubuntu Early Intervention Programme’ was the only support group intervention with explicit government involvement [43]. All other interventions were implemented by non-governmental organisations.

Most interventions included in our review were facilitated solely by allied health professionals (occupational therapists, physiotherapists, or social workers), expert parents or community volunteers (n = 21, 67.7%) [8,14,18,19,24–28,30,32–34,36,38–41,43,45,47] (**Table 3**). Three (9.7%) studies utilised a combination of medical specialist and allied health facilitators [31,37,42]. Two (6.5%) interventions were facilitated by psychologists [20,21]. Five (16.2%) studies did not specify the qualifications and skills of facilitators [22,23,29,35,44]. The qualifications and profession of facilitators did not appear to differ according to country income level. Three identified interventions utilised a cascade training model of facilitation [19,39,40]. 19 studies provided information regarding support group size. The mean support group size was 9.95 participants per facilitator, the median group size was eight to ten participants per facilitator. The support group sizes ranged from three to 25 participants.

#### Teaching methodology

Most interventions in our review included an element of group discussion (n = 17, 54.8%) [14,18–20,23–28,31,32,36,37,39,42,47]. Hands-on activities such as practicing parenting skills under supervision were also often utilised in interventions identified by our review (n = 11, 35.5%) [18,19,21,23,24,29,32,37,39] (**Table 3**).

#### Mode of delivery

Almost all studies included in our review involved in-person support groups, sometimes accompanied by home-visits [8,36,38,42,43,45] or telephone follow-up [24,38]. The purpose of home visits varied across interventions, however recurring themes include individualised goal setting, provision of follow-up training, engagement of other family members and tailored rehabilitation. One intervention was delivered via social media [19]. Almost half of the identified interventions included the child with disability in intervention activities (n = 14, 45%), typically through play therapy and practice of parent-child interventions under facilitator supervision.

### Outcomes of group-based caregiver support interventions

Studies included in our review used a variety of measurement tools (**Table 4**) and reported diverse outcomes. Of the 31 studies, 15 studies (48%) included a quantitative measure of intervention efficacy and most (n = 13) demonstrated significant improvements in at least one parental or child outcome measure. Three included randomised-controlled-trials (75%) demonstrated significant improvement in at least one parental or child outcome, which is comparable to findings from non-randomised-control-trials.

**Table 4:** Quantitative caregiver and child outcome measures used in studies on group-based caregiver support interventions

Outcome	Measures
Parent	
*Stress/stress*	PSI, BAI, PSS
*Coping*	CSI, MCSI
*Parenting skills*	KBMAQ, PS, PPC
*Depression*	HDRS-17, BDI, DASS
*Self-efficacy*	PSEM
*Quality of life*	PedsQLFMI, WHO-5
*Insight on child’s condition*	KCPQ, KLF
*Stigma*	ISE
*Empowerment*	FES
*Emotional burden*	FBAS-ID
*Hopelessness*	BHS
*Social support*	MSPSS
Child	
*Aggression*	ASIQ
*Self-harm*	VABS, ABC, ECBI
*Behaviour*	COPM
*Independent functioning*	GARS2, CARS, WHODAS-Child, DD-CDAS, CDP
*Condition severity*	GMFM-66, GMFCS
*Physical health*	CFCS, VSS
*Communication/speech*	CFCS, VSS
*Well-being*	SDQ

ABC – Autism Behaviour Checklist, ASIQ – Aggression and Self-Injury Questionnaire, BAI – Beck Anxiety Inventory, BDI – Beck Depression Inventory, BHS – Beck Hopelessness Scale, CARS – Childhood Autism Rating Scale, CDP – Communication Disability Profile, CFCS – Communication Function Classification System, COPM – Canadian Occupational Performance Measure, CSI – Coping Strategy Index, DASS – Depression Anxiety Stress Scale, DD-CDAS – Developmental Disorders-Children Disability Assessment Schedule, ECBI – Eyberg Child Behaviour Inventory, FBAS-ID – Family Burden Assessment Scale for Families with Children with Intellectual Disabilities, FES – Family Empowerment Scale, GARS2 – Gillam Autism Rating Scale, GMFCS – Gross Motor Function Classification System, GMFM-66 – Gross Motor Function Measure, HDRS-17 – Hamilton Depression Rating Scale, ISE – Inventory of Stigmatizing Experiences, KBMAQ – Knowledge of Behavioural Management of Aggression Questionnaire, KCPQ – Knowledge of Cerebral Palsy Questionnaire, MCSI – Modified Caregiver Strain Index, MSPSS – Multi-dimensional Scale of Perceived Social Support, PSEM – Parenting Sense of Efficacy Measure, *PedsQL*FMI – *PedsQL* Family Impact Model, PPC – Parent Problem Checklist, PS – Parenting Scale, PSI – Parent Stress Index, PSS – Parental Stress Scale, SDQ – Strengths and Difficulties Questionnaire, VABS – Vineland Adaptive Behaviour Scale, VSS – Viking Speech Scale, WHO5 – World Health Organisation Well-being Index, WHODAS – Child- World Health Organisation Disability Assessment Schedule for Children

#### Parental outcomes

Studies included in our review found significant improvements in caregiver coping skills [20,38], parenting skills [24,31] and insight on child’s condition [29,37] (**Table 5**). There were inconsistent findings on anxiety, depression, and quality-of-life (**Table 5**). One Turkish study reported an unexpected increase in caregiver emotional burden following participation in an educational intervention for caregivers of children with intellectual disability (three-week intervention addressing characteristics of intellectual disability, behavioural management, emotional regulation and communication through lectures, group discussion, role-playing and storytelling) [29]. Despite positive effects on parental outcomes not always being reflected quantitatively, caregivers reported many qualitative benefits from support-group interventions including increased social inclusion within the community [25,33,42], improved self-confidence in caretaking [27,34] and improved mental well-being [18,42,43].

**Table 5:** Effect of caregiver support group intervention on quantitative outcomes

Condition	Autism					Cerebral Palsy				Developmental disorders				Intellectual disability	General disability
**Intervention**	Pashazadeh Azari et al., 2019 [30]	Al-Khalaf et al., 2014 [20]	Bello-Mojeed et al., 2016 [31]	Bordini et al., 2020 [21]	Zu et al., 2019 [22]	Zuurmond et al., 2018 [8]	van Aswegen et al., 2019 [23]	Karim et al., 2021 [36]	Dambi et al., 2016 [37]	Muthukaruppan et al., 2020 [38]	Leung et al., 2013 [24]	Hamdani et al., 2015 [39]	Hamdani et al., 2021 [40]	Cenk et al., 2016 [29]	Bunning et al., 2020 [14]
**Outcomes**															
**Parent**															
Stress/anxiety		+*			0†		0†				+*				
Coping		+*								++‡					
Parenting skills			++‡								+				
Depression				0†	+*			0†							
Self-efficacy	+														
Quality of Life						++‡	0†					0†	+*		
Insight on child’s condition									++‡					++‡	
Stigma												+*	0†		
Empowerment										++‡		++‡ (Community involvement/service) 0(family)	0†		
Emotional burden														– –§	
Hopelessness														++‡	
Social Support															++‡
**Child**															
Aggression			++‡												
Self-harm			+*												
Behaviour				0†							+*				
Independent functioning	++‡														
Condition severity	++‡				+*							++‡	0†		++‡
Physical health						–‖		++‡							
Communication/speech								++‡							
Well-being													0†		
															

*Statistically significant (*P* < 0.05) positive effect (benefit) on measured outcome, i.e. improved coping or reduced depression, anxiety etc.

†No significant effect (neutral).

‡Statistically significant (*P* < 0.001) positive effect (benefit) on measured outcome, i.e. improved coping or reduced depression, anxiety etc.

§Statistically significant (*P* < 0.001) negative effect (detriment) on measured outcome, i.e. increased stress, anger etc.

‖Statistically significant (*P* < 0.05) negative effect (detriment) on measured outcome, i.e. increased stress, anger etc.

#### Child outcomes

Fewer studies included in this review reported on child outcomes. Of these, findings included a reduction in quantitative measures of children’s disability severity [14,22,30,39] and parental report of improved emotional and behaviour adaptation [18] and family relationships [42] (**Table 5**). One study identified increased stunting across the timespan of the support group intervention [8] (**Table 5**).

### Identified barriers and enablers of support group implementation

Half the included studies (n = 17, 55%) reported on barriers or enablers to intervention implementation (**Table 6**). The main identified barriers included time restraints for caregivers due to domestic and occupational responsibilities [27,28,33,34,39,42], lack of support from family members not involved in the intervention [18,24,34,42,43] and poverty [33,34,43,44]. Supportive facilitators [19,27,34,43] and caregiver solidarity through peer support [19,27,28,34] were identified as enablers of the success of support-group implementation in LMICs. Limited data suggests that highly relevant intervention content [43] and skilled knowledgeable facilitators [45] also contributed to the success of interventions.

**Table 6:** Identified barriers and enablers of intervention implementation

Theme	Barrier	Enabler
Accessibility of intervention	Inaccessibility of intervention at centralised clinic location due to lack of access to transport [43,69]	Accessible intervention location at community site [25,70]
Feasibility of intervention	Length of intervention not long enough to be impactful [18,69]	Interventions adapted to parental knowledge level [31]
	Low applicability of intervention due to heterogeneity of child’s disease [36]	Relevant content [43]
		Inclusion of clear goals for parental involvement [27]
Factors intrinsic to intervention participants	Limited parental knowledge of child’s condition and behavioural interventions at baseline [31,69]	
	Poor parental literacy [31]	
	Parental mental health issues [21]	
	Preconceived parental stigma towards child’s condition [18,19,69]	
	Caregiver burnout [33]	
Factors intrinsic to intervention facilitators	Community volunteer attrition [45]	Experienced intervention facilitators [45]
	Lack of specialist training and experience [19]	Good demeanour demonstrated by facilitators [19,27,34,43]
Socioeconomic factors	Lack of support from family members not enrolled in intervention [18,24,34,43,69]	Parental solidarity through peer support [19,27,28,34]
	Time restraints due to domestic and occupational responsibilities [27,28,33,34,39,69]	
	Lack of resources [45]	
	Poverty [33,34,43,44]	
Environmental factors	COVID-19 pandemic [18,19]	Strong relationships with health facilitators [45]
Political factors	Lack of government commitment to supporting children with disabilities [34,45]	Building strong relationship with government agencies (e.g. Ministry of Health) [45]
		Strong funding partnerships with government and other organisations [45]
Sociocultural factors	Stigma in society towards children with disability and their mothers [34]	

## DISCUSSION

This review identified and systematically described a small but important emerging literature regarding the design, implementation, and impact of caregiver support interventions for children with disabilities in LMICs. There were limited studies conducted in the lowest resourced settings, where there may be the greatest need for education and support interventions.

However, despite these limitations, studies included in this review provide important evidence to guide development and implementation of care and support for children with disabilities and their families in LMICs, as limited access to formal early childhood intervention services, education, health care, allied health, ancillary and social services in LMICs often leaves primary caregivers feeling alone and unsupported [8]. Family support is often the weakest part of health service delivery for children with disability, often consisting of little more than brief counselling or education [48–50]. In exploring the content, delivery, and implementation factors that may influence success, we highlight six key lessons for future implementation and scale-up.

### Lessons for future caregiver support interventions

First, the content of caregiver support interventions should be relevant and appropriate to participants and delivered in a way to promote individual and group learning and growth. Our findings regarding intervention content valued by caregivers are consistent with previous literature, which has shown that supporting ADLs in children with disability helps promote optimal child development by providing them with a sense of normality [3,23] and that effective management of challenging behaviours can improve the mental well-being of children with disability and their caregivers [31,51,52]. Behavioural problems are an important issue to address in neurodevelopmental disorders, as children with developmental disabilities are up to three times more likely to exhibit significant behaviour problems [53]. In a LMIC context, interventions should also aim to dispel myths regarding the origin of disability and alleviate feelings of blame felt by primary caregivers. Religion and superstition have a dominant influence on many South-East-Asian and African cultures, where childhood neurodevelopmental disability is often attributed to a curse from God or past personal failings [34,54,55]. Working with caregivers to reframe childhood disability can be beneficial towards caregiver and child well-being.

Second, caregiver support groups are an opportunity to expand social understandings of disability and challenge prejudicial social norms [8,14,22,25–29,33,34,38,39,44] and build caregivers’ psychosocial coping strategies [18,19,22,29,38,40,42,44,47]. Addressing caregiver and community understanding of disability promotes greater acceptance of children with disabilities in their families and the community, fostering a more positive environment for children with disabilities to prosper [3,43]. Whilst modules dedicated to parental upskilling and emotional well-being can combat the high prevalence of carer burnout in caregivers of children with disabilities and optimise parental health such that they can effectively care for their children [56].

Third, facilitators are crucial to successful implementation of caregiver support group interventions and while decentralisation and use of non-specialist facilitators is attractive for many reasons, they must be properly supported to avoid excessive volunteer attrition. Cascade facilitation models, in which a master trainer coaches less experienced trainers, who in turn pass on their knowledge to a larger group of community volunteers, have been used for community health promotion activities allowing for interventions to have broader reach and greater sustainability [19,29]. In LMIC settings, where access to specialist services is often limited, non-specialist intervention facilitation is essential for providing ongoing cost-effective accessible support to caregivers [8]. However, despite the high promise of non-specialist facilitators, intervention developers should also be mindful of community members’ lack of formal health care training and capacity for handling any emergencies such as concerns for child abuse [57]. Furthermore, targeted selection of facilitators with the most appropriate skillset to support specific aspects of children’s disability, such as the involvement of psychologists to address behavioural problems and physiotherapists to address physical rehabilitation can contribute to improved positive outcomes and acceptance of support interventions [36]. Regular upskilling workshops and ongoing supervision of intervention facilitators by a health care professional may also contribute to the long-term success of caregiver support interventions.

Fourth, some of the greatest benefits of support groups comes from active peer-to-peer learning [34]. This may be particularly important for stigmatised groups, such as those with disability, enabling caregivers to find comfort connecting with peers in similar circumstances and facilitating social inclusion [26,33,42]. Limited data suggests that remote intervention delivery may be possible and facilitates strong social connection despite the remote nature of delivery, with additional potential benefits to cost-efficacy and access but also real challenges – particularly regarding access to technology and digital literacy [19,58].

Fifth, the caregiver support interventions should be designed and implemented with the broader health and social structures in mind, and with a long view to sustainability. Many of the barriers and enablers identified in our review could be proactively addressed by building strong relationships with local service providers and community leaders and being transparent about ongoing government and funding support [3,45]. Currently few caregiver support interventions are implemented through non-government organisations. Implementation research in routine health services and cost-effectiveness analyses may be important for supporting further exploration of sustainability of caregiver support interventions in the future.

Sixth, LMICs are diverse in their cultures, thus interventions should be adapted for different cultural contexts to appropriately align with community values and lifestyle for maximal sustainability [59]. Testing of the feasibility and acceptability of culturally adapted interventions should also be undertaken when implementing pre-existing interventions in a new country. Successful scale-up of interventions in LMICs is influenced by multiple factors including effective leadership, the fiscal environment and community engagement [59–61]. Thus, it is integral to involve community leaders and local governments to ensure interventions align with pre-existing policies and systems [62].

### Measuring and understanding outcomes

The findings of generally beneficial effects of caregiver support group interventions, irrespective of geographical and socioeconomic strata, were consistent with studies in high-income regions [11,63].Group-based support interventions for caregivers with children with disabilities have the potential to improve child problem behaviour outcome measures and caregiver parenting skills and overall quality of life, which is consistent with findings of reviews conducted on caregiver support interventions for caregivers of children with CP and ASD conducted in HICs [12,64]. However, results from individual studies were often mixed and quantitative measures did not always reflect qualitative feedback from participants.

For example, in terms of caregiver outcomes the higher emotional strain caregivers reported in Cenk et al’s. (2016) study may have been caused by caregivers fully understanding the prognosis and consequences of their child’s disability following education on their child’s disability [29]. Zuurmond et al’s (2018) study also identified increased stunting in children with cerebral palsy following intervention participation. The authors suggested that this finding may be confounded by the natural progression of cerebral palsy [8].

These inconsistent and sometimes unexpected outcomes, highlight the importance of ongoing rigorous implementation research and intervention monitoring and evaluation to fully understand impact, including potential unintended consequences of caregiver support interventions across settings moving forward.

Currently, variable outcome measurement and limited description of intervention theories of change make it challenging to know how various caregiver support group interventions effect change for different populations and in different contexts.

Future implementation research will benefit from development of more standardised outcome measurement approaches, guided by relevant frameworks (e.g. the International Classification of Functioning Disability and Health for Child and Youth – ICF-CY and the Consolidated Advice for Reporting ECD implementation research (CARE) guidelines [65,66].

### Limitations

Longer duration follow-up for outcome measurement and cost-effectiveness data are also needed. Our database search was restricted to the English language for feasibility. Meta-analysis of outcomes was not undertaken due to the great heterogeneity of investigated primary outcomes in included studies. Most identified studies were qualitative and reflected a small sample size. Very few studies were RCTs and even fewer followed CONSORT guidelines [67]. We were unable to perform a meta-analysis due to great variability in studied outcome measures. Generalisability of findings from this review were reduced by the great variability in study methodology and sociocultural contexts in which interventions were implemented.

## CONCLUSIONS

A small but increasing body of literature demonstrates the benefits of group-based support interventions for caregivers of children with disabilities in LMICs in improving caregiver and child outcomes, as well as core intervention content, composition, and barriers and enablers to successful implementation. To better understand adaptation of caregiver support interventions across contexts as well as factors required for effective and sustainable impact at scale, further implementation research and intervention monitoring and evaluation is required including more clearly articulated intervention theory of change, greater standardisation of outcome measures, longer term follow-up and cost-effectiveness data to better inform policy and practice. Specifically, cohort studies investigating the long-term impact of interventions on caregivers of children living with disability and their communities may be beneficial.

## Additional material


Online Supplementary Document
