# Supplementary material for: Group-based caregiver support interventions for children living with disabilities in low-and-middle-income countries: Narrative review and analysis of content, outcomes, and implementation factors
Source: J Glob Health. 2024 Apr 12;14:04055. doi: 10.7189/jogh.14.04055 (PMC11009896; doi:10.7189/jogh.14.04055)
Supplement: Online Supplementary Document [file jogh-14-04055-s001.pdf]

Supplemental material

Composition, outcomes, and implementation of group education and support programs for caregivers of children with disabilities in lower-and-middle-income countries; a narrative review with systematic approach

Authors: Christine He[1], Natalie Evans[2], Hamish Graham[3], Kate Milner[4]

Contents

Table S1 Literature review full search ……… 2

Figure S1 Study characteristics ……6

Table S2 Content of psychosocial intervention programs for caregivers of children with disability in LMIC ………….. .9

[1] Universiy of Melbourne, Victoria, Australia

[2] Melbourne Children's Global Health, Murdoch Children's Research Institute, University of Melbourne

[3] Melbourne Children's Global Health, MCRI, University of Melbourne, Royal Children's Hospital, Melbourne, Australia.

[4] Neurodisability & Rehabilitation Research Group, Murdoch Children's Research Institute, University of Melbourne, Department of Pediatrics & Department of Neurodevelopment & Disability, Royal Children's Hospital, Melbourne, Australia

Search Strategy for identification of studies
The following databases were searched in April 2022 for references back to 2012: Medline (Ovid), Embase (Ovid) and PsycInfo (OVID) using thesaurus and keywords.

Thesaurus terms used:
Population:

a) Disability: *neurodevelopmental disorders/ or exp *"attention deficit and disruptive behavior disorders"/ or *child behavior disorders/ or exp *child development disorders, pervasive/ or exp *communication disorders/ or *developmental disabilities/ or *intellectual disability/ or exp *learning disabilities/ or *motor skills disorders/ or *mutism/ or *reactive attachment disorder/ or exp *tic disorders/ or neurocognitive disorders/ or exp *cognition disorders/ or *brain injuries/ or exp *brain hemorrhage, * traumatic/ or exp *brain injuries, traumatic/ or exp *brain injury, chronic/ or *epilepsy, post-traumatic/ or exp *Stroke/ or *Cerebral Palsy/ or exp *movement disorders/ or *spinal cord diseases/ or *amyotrophic lateral sclerosis/ or *spinal cord compression/ or exp *spinal cord injuries/ or exp *spinal cord vascular diseases/ or exp *Congenital Abnormalities/ or exp *Muscular Dystrophies/ or exp *Muscular Atrophy, Spinal/ or exp *Spinal Dysraphism/ or *Cystic Fibrosis/exp *Multiple Sclerosis/ or exp *Epilepsy/ or exp *seizures / or exp *Dwarfism/ or exp *Child Development Disorders, Pervasive/ or *trisomy 18 syndrome/ or *turner syndrome/ or Fetal Alcohol Spectrum Disorders/ or exp *Hearing Loss / or *sensation disorders/ or *disabled persons/ or *disabled children/ or *persons with hearing impairments/ or *visually impaired persons/
b) Caregiver: exp *parents/ or *Caregivers/ or *Legal Guardians

Intervention: exp * Social Support/

Keywords:
Population:

a) disability (disabilit* or functional-impairment* or deficit or neurodevelopmental-disorder* or brain-injur* or stroke or hypoxic-ischaemic-encephalopath* or cerebral-palsy or movement-disorder or paralysis or congenital-abnormal* or muscular-dystroph* or Spinal-muscular-atroph* or movement-disorder* or amyotrophic-lateral-sclerosis or spinal-cord-disease or spinal-cord-compress*OR spinal-cord-vascular-disease or spinal dysraph* or spina-bifid* or myelomeningocele or cystic-fibrosis or multiple-sclerosis or epilep* or dwarf* or achondroplas* or intellectual-disab* or attention-defecit or developmental-delay or learning-disab* or autis* or asperger* or child-behaviour-disord* or chiled-development-disord* or development-disab* or motor-skill-disord* or mutism* or fragile-X* or prada-willi* or angelman* or down-syndrome* or trisomy* or edwards-syndrome or patau-syndrome or fetal-alcohol-spectrum-disorder* or

intellectual-impair* or learning-impair* or Autis* or tourette* or tics ordeaf* or blind* or visual-impair* or hearing-impair* or visual-impair* or low-vision or hearing-loss or auditory-impair* or sensory-processing-disorder*).tw,kf.

b) Caregiver (father* or mother* or caregiver* or care-giver*or guardian*).tw.kf

Intervention: ((support or training or group or community or educat*) adj3 (program* or intervention* or therapy or self-help or psychological or wellbeing or peer)).tw,kf,hw.

Age limits:
newborn* OR new-born* OR baby OR babies OR neonat* OR neo-nat* OR infan* OR toddler* OR pre-schooler* OR preschooler* OR kinder OR kinders OR kindergarten* OR kinder-aged OR boy OR boys OR girl OR girls OR child OR children OR childhood OR pediatric* OR paediatric* OR school- age* OR schoolage* OR schoolchild* OR schoolgirl* OR schoolboy* OR adolescen* OR youth OR youths OR teen OR teens OR teenage*

LMIC filter:

1. developing countries/
2. (austere or (limited adj2 resource*) or (low adj2 resource*) or (transitioning adj econom*) or (third adj world) or LMIC or LMICs or (lami adj countr*) or (transitional adj countr*) or (low adj gdp) or (low adj gnp) or (low adj gross adj domestic) or (low adj gross adj national) or ((emerging or developing or (low adj income) or (middle adj income) or (low adj3 middle) or underdeveloped or under-developed or (less* adj developed) or underserved or under-served or deprived or poor*) and (countr* or nation*1 or econom* or population or world))).tw,kf.
3. exp africa/
4. americas/ or exp caribbean region/ or exp central america/ or latin america/ or mexico/ or exp south america/
5. europe/ or exp europe, eastern/ or exp transcaucasia/
6. antarctic regions/ or exp atlantic islands/ or exp indian ocean islands/ or exp pacific islands/
7. New Guinea/
8. asia/ or exp asia, central/ or asia, southeastern/ or borneo/ or cambodia/ or east timor/ or indonesia/ or laos/ or malaysia/ or mekong valley/ or myanmar/ or philippines/ or thailand/ or vietnam/ or asia, western/ or bangladesh/ or bhutan/ or india/ or middle east/ or afghanistan/ or iran/ or iraq/ or jordan/ or lebanon/ or oman/ or saudi arabia/ or syria/ or turkey/ or yemen/ or nepal/ or pakistan/ or sri lanka/ or far east/ or china/ or tibet/ or exp korea/ or mongolia/
9. (Afghanistan or Albania or Algeria or Angola or Antigua or Argentina or Armenia* or Aruba or Azerbaijan or Bahrain or Bangladesh or Barbados or Barbuda or Belarus or Byelarus* or Byelorussian or Belorussian or Belorus* or Belize or Benin or Bhutan or Bolivia or Bosnia or Botswana or Brasil or Brazil or Bulgaria or (Burkina adj Fas*) or (Upper adj Volta) or Burma or Burundi or Cambodia or Khmer

or Kampuchea or Cameron* or Cameroon* or (Cape adj Verde) or (Cabo adj Verde) or (Central adj African adj Republic) or Chad or Chile or China or Colombia or Comoros or (Comoro adj Island*) or Comores or Mayotte or Congo or Kongo or (Cook adj Island*) or (Costa adj Rica) or (Cote adj D'ivoire) or Croatia or Cuba or Cyprus or Czech* or Djibouti or Dominica or Dominican or (East adj Timor) or (East adj Timur) or Ecuador or Egypt or El-Salvador or (Equatorial adj Guinea) or Eritrea or Estonia or Ethiopia or Fiji or (French adj Somaliland) or Futuna or Gabon or (Gabonese adj Republic) or Gambia or Gaza or (Georgia* adj Republic) or Ghana or Grenada or Guam or Guatemala or Guinea or Guiana or Guyana or Haiti or Herzeg* or Hercegovina or Honduras or Hungary or India or Indonesia or Iran or Iraq or (Ivory adj Coast) or Jamaica or Jordan or Kazakh* or Kenya or Kiribati or Korea or Kosovo or (Kyrgyz adj Republic) or Kyrgyzstan or Kirghizia or Kirghiz or Kirgizstan or Laos or (Lao* adj2 Democratic adj Republic) or (Lao* adj PDR) or Latvia or Lebanon or Lesotho or Basutoland or Liberia or Libya or Lithuania or Macedonia or Madagascar or (Magalasy adj Republic) or Malawi or Malay* or Sabah or Sarawak or Maldives or Mali or (Marshall adj Island*) or Mauritania or Mauritius or (Agalega adj Island*) or Mexico or Micronesia or Moldov* or Mongolia or Montserrat or Montenegro or Morocco or Ifni or Mozambique or Myanma* or Namibia or Nauru or Nepal or (Netherlands adj Antilles) or (Dutch adj Antilles) or (New adj Guinea) or (New adj Caledonia) or Nicaragua or Niue or Niger or Nigeria or (Northern adj Mariana adj Island*) or Nyasaland or Oman or Pakistan or Palau or Panama or (Papua adj New adj Guinea) or PNG or Palestine or Paraguay or Peru or Philipines or Philippines or Phillipines or Phillippines or Poland or (Puerto adj Rico) or Yemen or Romania or Roumania or Rumania or Russia* or Rwanda or Ruanda or (Saint adj Kitts) or (St adj Kitts) or Nevis or (Saint adj Vincent) or (St adj Vincent) or Grenadines or Samoa* or (Navigator adj Island*) or (Saint adj Lucia) or (St adj Lucia) or (Saint adj Helena) or (St adj Helena) or (Sao adj Tome) or (Saudi adj Arabia) or Senegal or Serbia or Seychelles or (Sierra adj Leone) or Slovenia or Slovak* or (South adj Africa) or (Solomon adj Island*) or Somalia or (Sri adj Lanka) or Ceylon or Sudan or Surinam* or Swaziland or Eswatini or Syria or Syrian-Arab-Republic or Tajikistan or Tadzhikistan or Tadjikistan or Tadzhik or Tanzania or Thailand or Tibet or Timor-Leste or Togo or (Togolese adj Republic) or Tokelau or Tonga or Trinidad or Tobago or Tunisia or Turkey or Turkmenistan or Turkmen or Tuvalu or Uganda or Ukraine or Uruguay or Urundi or USSR or (Soviet adj Union) or "Union of Soviet Socialist Republics" or Uzbekistan or Vanuatu or (New adj Hebrides) or Venezuela or Vietnam or (Viet adj Nam) or (Wallis adj2 Futuna) or (United adj Arab adj Republic) or (West adj Bank) or (West adj Indies) or Yemen or Yugoslavia or Zaire or Zambia or Zimbabwe or Rhodesia).tw,kf.

10. (africa or americas or caribbean or (central adj America) or (latin adj America) or (south adj America) or (eastern adj Europe) or Transcaucasia or antarctic or (atlantic adj island*) or (indian adj ocean adj island*) or (pacific adj island*) or polynesia or (central adj asia) or (southeast* adj asia) or (south-east* adj asia) or borneo or mekong or (western adj asia) or (middle adj east) or (far adj east)).tw,kf.
11. 1 or 2 or 3 or 4 or 5 or 6 or 7 or 8 or 9 or 10

In summary, the search terms for Population, Intervention, Age limits and LMIC filter were combined using the Boolean ‘AND’, i.e., Population AND Intervention AND Age limits AND LMIC filter.

Results were limited to English language and child 0-18 years.

We also reviewed reference lists of related systematic and other reviews, or studies. We did not identify any relevant studies outside our search on Medline, Embase and PubMed

During the screening stage of our literature review, we refined our search strategy to exclude epilepsy, seizure disorders and disordered behaviour.

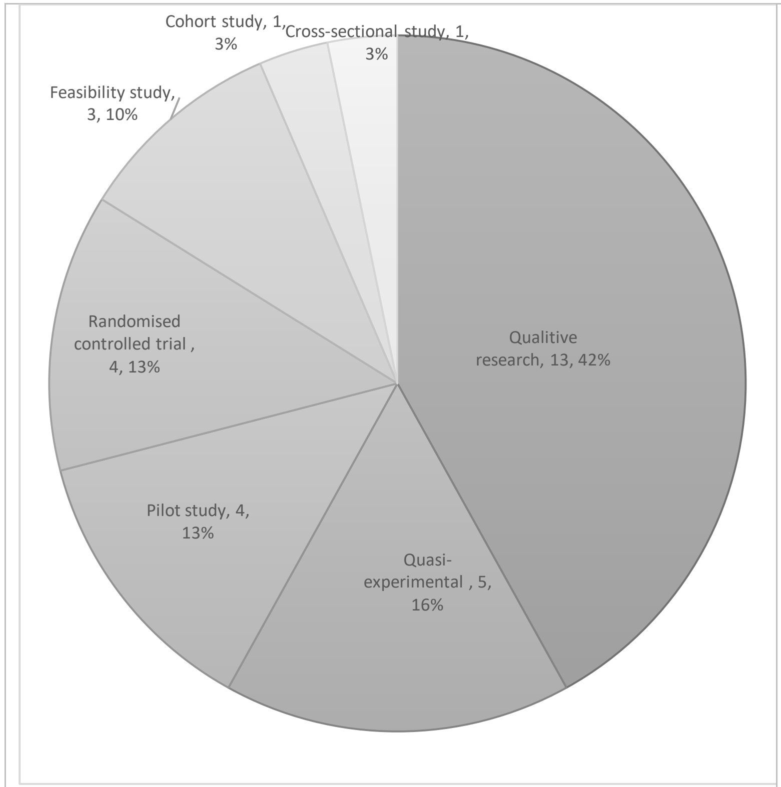

Figure S1a. Study design

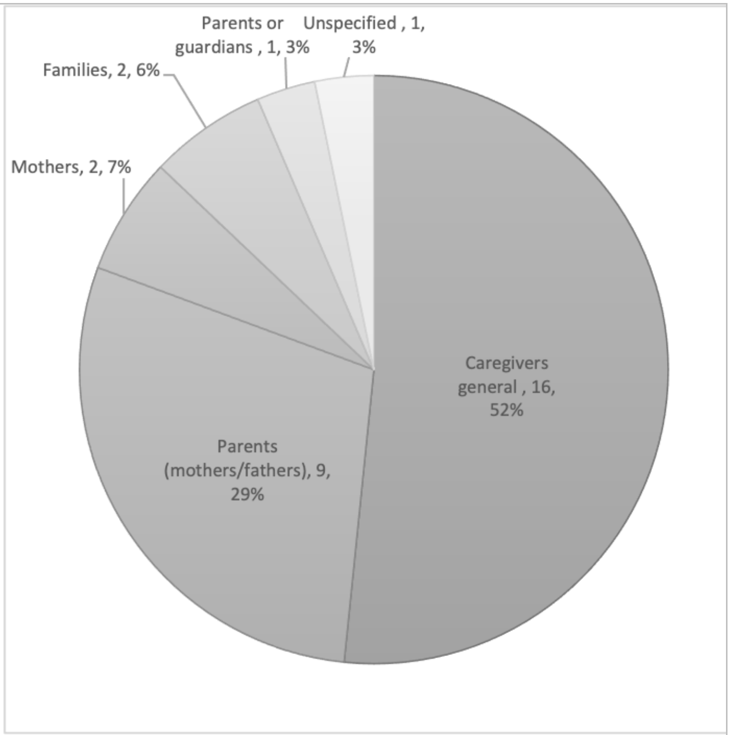

Figure S1b. Target population

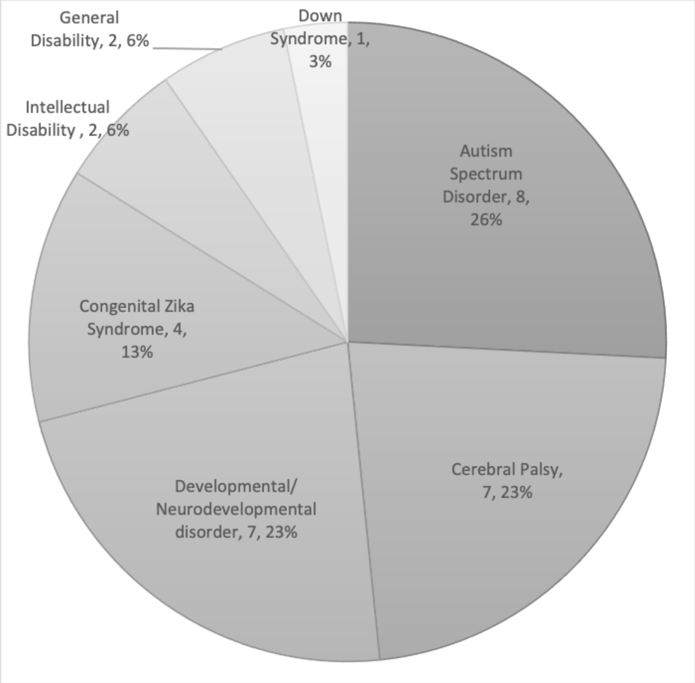

Figure S1c. Condition of Child

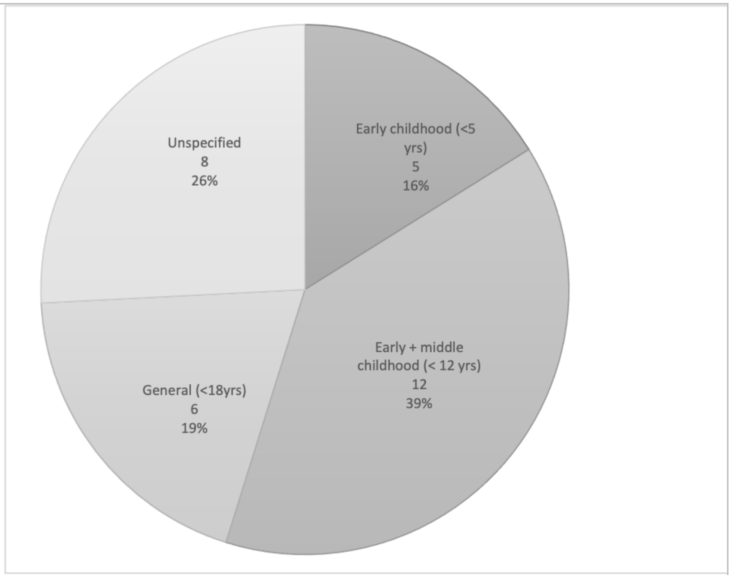

Figure S1d. Age of Child

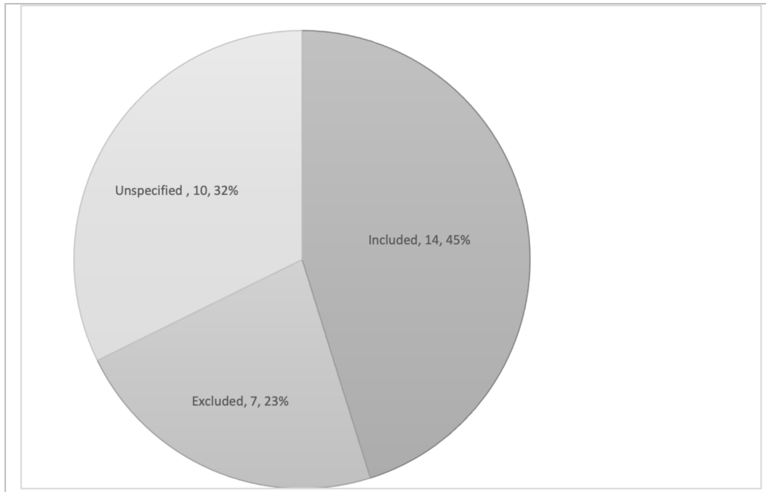

Figure S1e. Child involvement in intervention

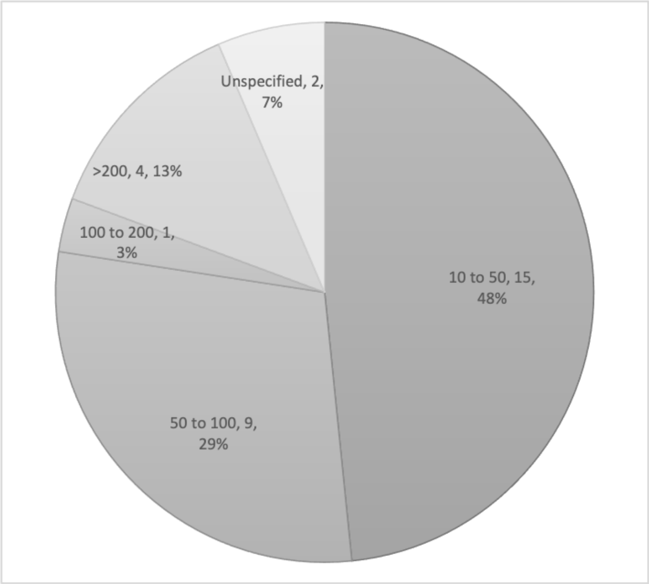

Figure S1f. Sample size

Figure S1. Study Characteristics

Table.S2 Content of psychosocial intervention programs for caregivers of children with disability in LMIC

	Content Domain																						
**Intervention**	**Behavioural management**	**Information about child`s condition**	**Child ADLS (Activities of Daily Living)/Health/ Function**	**Parenting Skills / caring for child with disability.**	**Communication**	**Child development**	**Play & Leisure**	**Parental stress reduction/ mindfulness/ emotional regulation**	**Peer Support**	**Future Planning**	**Management of medical complications**	**Child emotional regulation**	**Navigating community resources (education/disability/rights/ healthcare)**	**Advocacy**	**Parental expectations**	**Feeding / Nutrition**	**Community Inclusion**	**Child social skills**	**Movement/mobility**	**Child cognition**	**Child safety**	**Economic empowerment**	**Content not specified.**
**Autism Spectrum disorder**																							
SREIA (Stars and Rain Education Institute for Autism) (17)	X				X	X	X	X		X													
PET (Parent education and training program) (18)	X		X	X				X			X												
CI-ASD (Contextual intervention adapted for autism spectrum disorders) (29)	X		X						X			X											

PCTP (Parent-Child Training Program) (31)																							X
Unnamed Jordan ASD specific intervention (19)	X	X			X				X														
Unamed Nigeria ASD specific intervention (30)	X	X																					
Unamed Brazil ASD specific intervention (20)				X																			
Unamed China ASD specific intervention (21)	X	X	X		X			X	X		X	X	X	X	X								
**Cerebral Palsy**																							
GTKCP (Getting to Know Cerebral Palsy) (7) (32) (33) (34)			X	X	X		X						X	X		X	X						
Hambisela (22)		X	X	X	X	X	X									X							

Unnamed Bangladesh CP specific intervention (35)			X		X	X			X									X	X	X			
Unnamed Zimbabwe CP specific intervention (36)		X							X														
Neurodevelopmental disorder																							
WHO CST (World Health Organisation Caregiver Skills Training) (45)	X		X		X		X	X			X												
Adapted WHO CST intervention Ethiopia. (46)	X		X	X	X	X		X												X			
Baby Ubuntu early intervention programme (42)		X	X	X	X		X									X	X		X				
mVBR-EI (Mobile Village Based Rehabilitation-Early Intervention)	X	X		X				X						X			X						

(37)																							
Triple P (23)	X	X		X											X								
Unnamed Pakistan neurodevelop mental disorder intervention (38)			X	X		X								X		X	X						
Unnamed Pakistan neurodevelop mental disorder intervention (39)	X	X	X				X	X	X		X												
**Congenital Zika Syndrome**																							
Juntos (24)(45) (26) (27)	X	X	X	X	X	X	X						X	X		X	X		X				
**Intellectual disability**																							
Titukulane (43)	X					X		X	X				X	X	X		X						
Unnamed Turkey Intellectual disability	X	X	X		X			X			X			X							X		

specific intervention (28)																							
**Non-specific disability**																							
Kusamala+ (44)			**X**			**X**										**X**							
Unnamed Kenya general disability intervention (13)									**X**				**X**	**X**			**X**					**X**	
**Down Syndrome**																							
PEP (Parent Empowerment Program) (40)			**X**				**X**																
